# Papillary thyroid cancer located in malignant struma ovarii with omentum metastasis: a case report and review of the literature

**DOI:** 10.1186/s12957-016-0776-x

**Published:** 2016-01-20

**Authors:** Yi Zhu, Chang Wang, Guo-Nan Zhang, Yu Shi, Shi-Qiang Xu, Shi-Jun Jia, Rong He

**Affiliations:** 1 Department of Gynecological Oncology, Sichuan Cancer Hospital, No. 55, Section 4, South People’s Road, Chengdu, 610041 Sichuan China; 2 Department of Ultrasound, Sichuan Cancer Hospital, Chengdu, Sichuan China; 3 Department of Gynaecology and Obstetrics, Chengdu First People’s Hospital, Chengdu, Sichuan China; 4 Department of Pathology, Sichuan Cancer Hospital, Chengdu, Sichuan China

**Keywords:** Struma ovarii, Papillary thyroid cancer, Metastases

## Abstract

**Background:**

The present of malignant transformation in struma ovarii is exceedingly rare. Malignant struma ovarii is usually asymptomatic and infrequently diagnosed preoperatively. Because of its rarity, there is no consensus about diagnosis and management in the literature.

**Case presentation:**

A 40-year-old female presented for her obstetric examination with an incidental finding of a pelvic mass. Patient was asymptomatic at presentation. A follow-up ultrasound confirmed the presence of a 3-cm mass in the left adnexa. Patient underwent a cytoreductive surgery (hysterectomy, bilateral salpingectomy and oophorectomy, omentectomy, appendectomy, and pelvic lymphadenectomy). Histopathology revealed a malignant struma ovarii with a focus of papillary thyroid carcinoma and the omentum metastasis. The patient with stage FIGO IIIc received 6 cycles of paclitaxel/carboplatin regimen after surgery. The patient subsequently had a thyroid scan that was normal with normal thyroid function. At a follow-up of 12 months, she is alive, in good clinical condition, and disease-free.

**Conclusions:**

Because of the rarity of these tumors and their lack of firm prognostic factors, treatment decisions should be made individually, based on pathologic and clinical parameters.

## Background

The majority of germ cell tumors are mature cystic teratomas, which account for approximately 15–20 % of all ovarian tumors [1]. Of these, 15 % contain thyroid tissue [2, 3]. Struma ovarii is diagnosed when thyroid tissue comprises more than 50 % of the teratoma [4, 5]. Struma ovarii accounts for only 2 % of all mature teratomas, and less than 5 % of struma ovarii present malignant transformation [4, 6, 7]. Owing to malignant struma ovarii (MSO) rarity, there has been some controversy about the diagnosis and treatment, and prognosis is difficult to evaluate.

We report an unusual case of papillary thyroid cancer in MSO, with metastasis to the omentum. Here, we presented a review of literature including the clinicopathologic features, differential diagnosis, and management.

## Case presentation

A 40-year-old female presented with an incidental finding of a pelvic mass during her obstetric examination before 6 years. A palpable mass was noted in the left-sided pelvis on physical examination. The patient did not have special symptoms and ascites. Abdominal ultrasonography (Voluson S8, General Electric Company, USA) revealed a 3.0 × 3.1 cm mixed echogenicity mass in the left adnexa, suggestive of an ovarian teratoma. The data obtained by routine blood, urine, and thyroid function tests, as well as tumor markers, were in the normal range. The patient had no history of thyroid dysfunction and no family history of ovarian or endocrine tumors. The patient had no relevant past interventions. Malignancy could not be excluded.

**Fig. 1:**
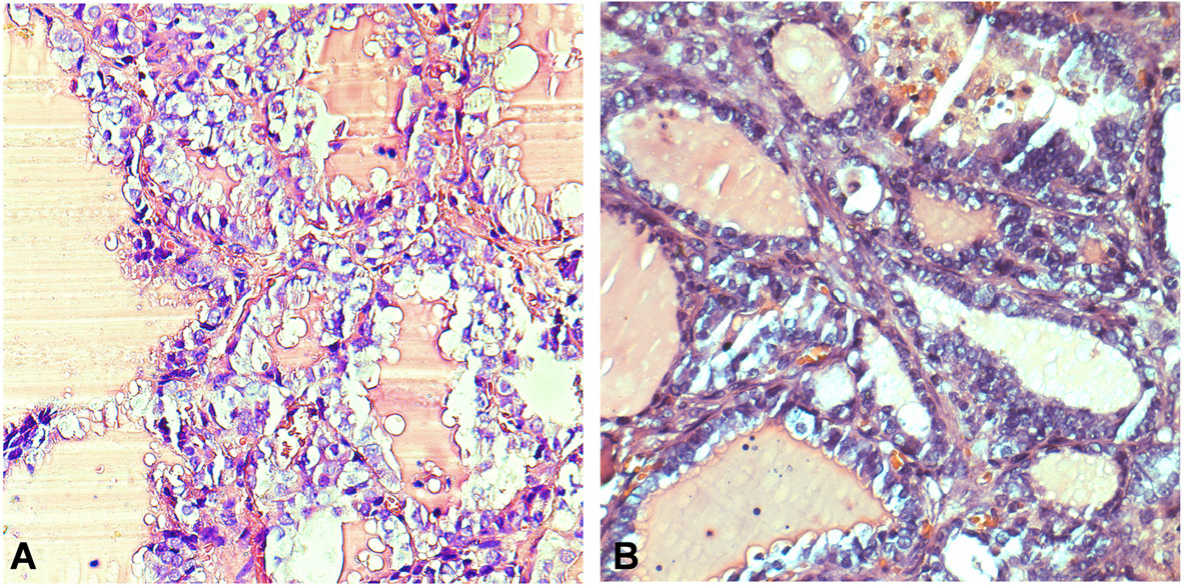
H&E staining of malignant struma ovarii tissues. H&E staining (**a**) showed fibrous cores of tissue lined by neoplastic cells which contain large overlapping “ground-glass” nuclei characteristic of thyroid papillary carcinoma. **b** Metastasis to omentum is shown (H&E, original magnifications ×400)

**Fig. 2:**
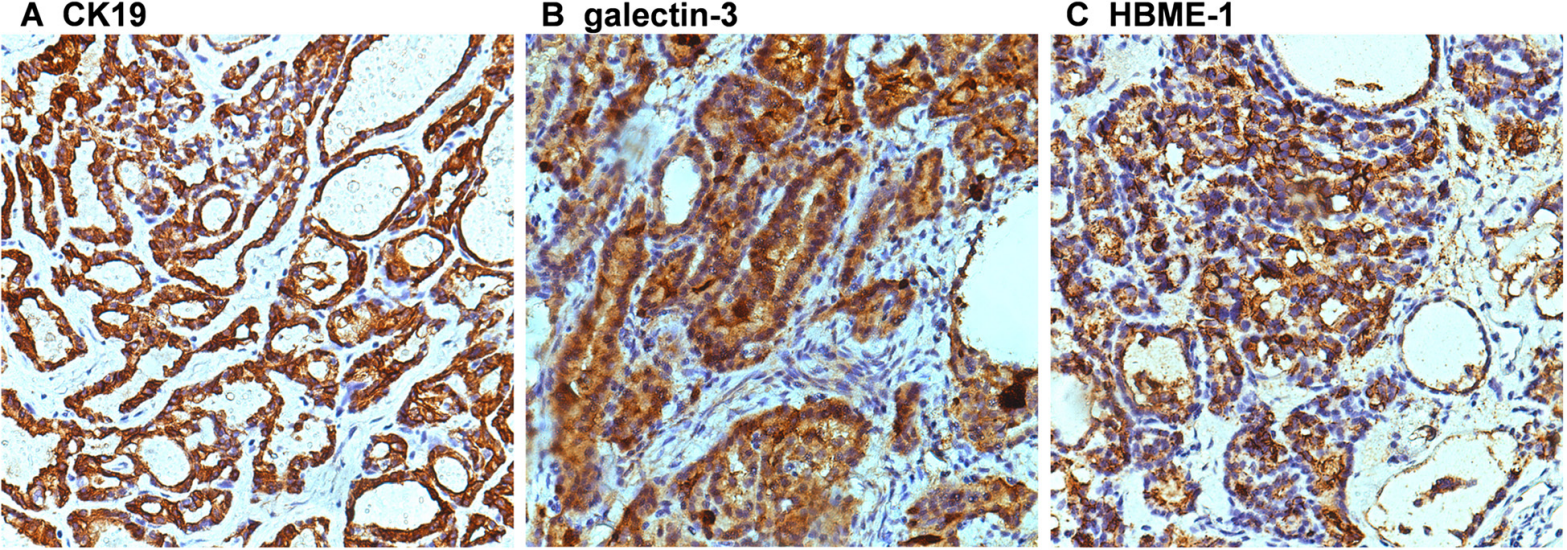
Immunohistochemical (CK19, galetin-3, HBME-1) staining of malignant struma ovarii tissues. **a** CK19 was diffuse and strong cytoplasmic staining in MSO. **b** MSO showing strong cytoplasmic staining of galectin-3. **c** MSO with beautiful complex papillae exhibits a HBME-1 membrane staining pattern (immunohistochemistry, original magnifications ×400)

The patient underwent laparoscopic ovarian cyst divesting surgery. At exploration, the uterus, right ovary, and fallopian tube were normal. The left ovary was enlarged, and a benign-looking white and smooth ovarian cystic mass was noted. Upon sectioning, the ovarian mass was partially cystic and grossly resembled papillary tumor. The peritoneal washing was negative on cytological examination. An intra-operative frozen and permanent paraffin section was proved to be a malignant struma ovarii with multiple foci of papillary thyroid carcinoma (Fig. 1a). In the papillary thyroid carcinoma focus, papillary thyroid tissue with increased mitotic activity and ground glass nuclei, intra-nuclear inclusions were detected. Also, a few psammoma bodies were identified. The tumor was restricted within the capsule, and vascular invasion was not identified. Immunohistochemically, tumor cells were strongly positive for thyroglobulin (Tg), thyroid transcription factor-1 (TTF-1), galetin-3, cytokeratin-19 (CK19), and human bone marrow endothelial cell-1 (HBME-1) (Fig. 2). Based on all these findings, the diagnosis was papillary thyroid cancer arising within malignant struma ovarii. Thus, 1 week later, the patient underwent a cytoreductive surgery (hysterectomy, bilateral salpingectomy and oophorectomy, omentectomy, appendectomy, and pelvic lymphadenectomy). The pathologic examination showed no metastatic neoplasm was found, except on the omentum material (Fig. 1b). After surgery, the patient with stage FIGO IIIc received 6 cycles systemic chemotherapy of paclitaxel (175 mg/m^2^; over 3 h intravenous infusion) plus carboplatin (area under the curve [AUC] = 5; over 1 h intravenous infusion), given on day 1 of a 21-day cycle. Patient subsequently had a thyroid scan that was normal with normal thyroid function.

### Discussion

Struma ovarii is a teratoma in which thyroid tissue is present exclusively or forms a grossly recognizable component of a more complex teratoma [8]. The rate of malignant transformation in struma ovarii is extremely low. The mean age at diagnosis of MSO was 43 years old [6, 9]. Most cases of MSO are subclinical. Clinical hyperthyroidism manifestations are seen in 5–8 % of the cases [10]. Moreover, insufficient concordance between morphologic features and clinical outcome in MSO is striking, making the behavior of these tumors particularly unpredictable [11]. It is reported that *BRAF V600E* gene mutations, a common pathogenesis for all papillary thyroid cancers, regardless of body location, were present in two thirds of MSO with papillary features [12].

The predominant sites of metastasis were adjacent pelvic structures, including the contralateral ovary; hematologic dissemination includes metastases of the lung, bone, liver, and brain, but metastasis is rare in patients with MSO. In this case, the patient had omentum metastasis. The reason is that the tumor can spread via regional lymphatics to pelvic and paraaortic lymph nodes and can directly infiltrate the omentum [11, 13].

Since it is a rare type of germ cell tumor encountered, there are no uniform diagnostic criteria for MSO. MSO is most often diagnosed postoperatively. The histopathological diagnosis adheres to the same criteria used for thyroid carcinoma: “ground glass” overlapping nuclei and nuclear grooves, or mitotic activity and vascular invasion [4]. Eighty-five percent of papillary carcinomas with the characteristics are “ground glass” nuclei. All pathologic patterns of thyroid gland malignancy may be found in struma ovarii, and papillary carcinoma is the most common [14–16]. Immunohistochemical staining with Tg, HBME-1, and galectin-3, often positive in papillary thyroid carcinoma, can also help confirm the diagnosis and are necessary for differential diagnosis from carcinoid and granulosa cell tumors [17, 18].

The absence of a primary lesion in the thyroid is necessary to exclude metastatic thyroid carcinoma to the ovary [4, 11]. The differential diagnosis from thyroid-type cancers from the thyroid and ovary depends on immunohistochemical stains for CK19 and TTF-1, supporting the independent existence of two cancers. Besides family history and a physical examination followed by an ultrasound of the thyroid gland, it is reported that unique characteristics on ovarian magnetic resonance imaging (MRI) might help to differentiate the two diseases.

The optimal treatment regimen for MSO is currently debated due to the difficulty to distinguish between benign and malign struma ovarii. Most authors advocate for an aggressive treatment based on TAH/BSO, lymph-node dissection and omentectomy, followed by adjuvant therapy, including external radiotheraphy, chemotheraphy, and thyroid suppression, regardless of the presence of distant metastases at time of diagnosis.

MSO might be over-treated, and this might impact long term the quality of life and fertility of affected women. Thus, conservative treatment such as unilateral oophorectomy should be the choice for patients who wish to fertility preservation, if pelvic imaging and surgical exploration do not reveal extra-ovarian disease [9, 19].

In addition, on the basis of the similarities between MSO and thyroid carcinoma, near-total thyroidectomy followed by radioactive iodine (I^131^) should be first-line therapy for patient with a higher risk of recurrence or with palindromia [12]. Treatment with thyroxine was not documented consistently enough to determine its impact on recurrence rates.

Patients with MSO had an excellent disease-specific survival rate, regardless of the management strategy employed. In 2015, Adam et al. published a study that included 68 patients with MSO [9]. The data indicated that overall survival rates for all patients were 94.3 % at 10 years and 84.9 % at 20 years, which was in agreement with the finding by Robboy et al. published in 2009 [20]. After TAH/BSO and chemotherapy, our patient is currently disease-free for the past 1 year. But long-term follow-up with thyroglobulin levels is necessary due to reports of increasing recurrence rates.

## Conclusions

Based on the rarity of these tumors and their lack of firm prognostic factors, treatment decisions should be made individually, based on pathologic and clinical parameters.

### Consent

Written informed consent was obtained from the owner of the patient for the publication of this case presentation and any accompanying images. A copy of the written consent is available for review by the Editor-in-Chief of this journal.

## Abbreviations

MSO: malignant struma ovarii
Tg: thyroglobulin
TTF-1: thyroid transcription factor-1
CK19: cytokeratin-19
HBME-1: human bone marrow endothelial cell-1
TP: paclitaxel/carboplatin
MRI: magnetic resonance imaging

## References

[1] Willemse PH, Oosterhuis JW, Aalders JG, Piers DA, Sleijfer DT, Vermey A (1987). Malignant struma ovarii treated by ovariectomy, thyroidectomy, and 131I administration. Cancer.

[2] Kabukcuoglu F, Baksu A, Yilmaz B, Aktumen A, Evren I (2002). Malignant struma ovarii. Pathol Oncol Res.

[3] Zakhem A, Aftimos G, Kreidy R, Salem P (1990). Malignant struma ovarii: report of two cases and selected review of the literature. J Surg Oncol.

[4] Devaney K, Snyder R, Norris HJ, Tavassoli FA (1993). Proliferative and histologically malignant struma ovarii: a clinicopathologic study of 54 cases. Int J Gynecol Pathol.

[5] Navarro MD, Tan MA, Lovecchio JL, Hajdu SI (2004). Case report: malignant struma ovarii. Ann Clin Lab Sci.

[6] DeSimone CP, Lele SM, Modesitt SC (2003). Malignant struma ovarii: a case report and analysis of cases reported in the literature with focus on survival and I131 therapy. Gynecol Oncol.

[7] Matsuda K, Maehama T, Kanazawa K (2001). Malignant struma ovarii with thyrotoxicosis. Gynecol Oncol.

[8] Scully RE (1970). Recent progress in ovarian cancer. Hum Pathol.

[9] Goffredo P, Sawka AM, Pura J, Adam MA, Roman SA, Sosa JA (2015). Malignant struma ovarii: a population-level analysis of a large series of 68 patients. Thyroid.

[10] Schmidt J, Derr V, Heinrich MC, Crum CP, Fletcher JA, Corless CL (2007). BRAF in papillary thyroid carcinoma of ovary (struma ovarii). Am J Surg Pathol.

[11] Rosenblum NG, LiVolsi VA, Edmonds PR, Mikuta JJ (1989). Malignant struma ovarii. Gynecol Oncol.

[12] Doganay M, Gungor T, Cavkaytar S, Sirvan L, Mollamahmutoglu L (2008). Malignant struma ovarii with a focus of papillary thyroid cancer: a case report. Arch Gynecol Obstet.

[13] Chan SW, Farrell KE (2001). Metastatic thyroid carcinoma in the presence of struma ovarii. Med J Aust.

[14] Gonzalez-Angulo A, Kaufman RH, Braungardt CD, Chapman FC, Hinshaw AJ (1963). Adenocarcinoma of thyroid arising in struma ovarii (malignant struma ovarii). Report of two cases and review of the literature. Obstet Gynecol.

[15] Culine S, Lhomme C, Farhat F, Droz JP (1995). Salvage chemotherapy in non-dysgerminomatous germ cell tumours of the ovary. Eur J Cancer.

[16] Szyfelbein WM, Young RH, Scully RE (1995). Struma ovarii simulating ovarian tumors of other types. A report of 30 cases. Am J Surg Pathol.

[17] Nakamura N, Erickson LA, Jin L, Kajita S, Zhang H, Qian X (2006). Immunohistochemical separation of follicular variant of papillary thyroid carcinoma from follicular adenoma. Endocr Pathol.

[18] Casey MB, Lohse CM, Lloyd RV (2003). Distinction between papillary thyroid hyperplasia and papillary thyroid carcinoma by immunohistochemical staining for cytokeratin 19, galectin-3, and HBME-1. Endocr Pathology.

[19] Shaco-Levy R, Peng RY, Snyder MJ, Osmond GW, Veras E, Bean SM (2012). Malignant struma ovarii: a blinded study of 86 cases assessing which histologic features correlate with aggressive clinical behavior. Arch Pathol Lab Med.

[20] Robboy SJ, Shaco-Levy R, Peng RY, Snyder MJ, Donahue J, Bentley RC (2009). Malignant struma ovarii: an analysis of 88 cases, including 27 with extraovarian spread. Int J Gynecol Pathol.

